# The Composition and Function of Bacterial Communities Associated with the Northern Root-Knot Nematode (*Meloidogyne hapla*) Populations Showing Parasitic Variability

**DOI:** 10.3390/microorganisms13030487

**Published:** 2025-02-22

**Authors:** Isaac Lartey, Gian M. N. Benucci, Terence L. Marsh, Gregory M. Bonito, Haddish Melakeberhan

**Affiliations:** 1 Agricultural Nematology Laboratory, Department of Horticulture, Michigan State University, East Lansing, MI 48824, USA; larteyis@msu.edu; 2 Department of Plant, Soil, and Microbial Sciences, Michigan State University, East Lansing, MI 48824, USA; benucci@msu.edu (G.M.N.B.); bonito@msu.edu (G.M.B.); 3 Department of Microbiology and Molecular Genetics, Michigan State University, East Lansing, MI 48824, USA; marsht@msu.edu

**Keywords:** nematode-microbe interaction, parasitic variability, root-knot nematode, microbiome

## Abstract

The co-existence of microbial communities and *Meloidogyne hapla* populations showing high, medium, and low levels of parasitic variability (PV) in mineral and muck soils with different soil health conditions in Michigan vegetable production fields is established. However, if PV relates or not to bacterial communities is unknown. This study characterized bacterial communities present on and in the body of nine *M. hapla* field and greenhouse sub-populations isolated from the mineral and muck fields. We utilized a high throughput sequencing of 16S rDNA. Results showed a variable composition (or abundance) of 65 genera in the field and 61 genera in the greenhouse isolates, with 12 genera of unknown and the rest belonging to 14 known functional groups. The medium- and low-PV populations shared more bacterial composition than either one with the high-PV population. Thus, laying a foundation for an in-depth understanding of if the observed associations have any role in cause-and-effect relationships with *M. hapla* PV.

## 1. Introduction

The northern root-knot (*Meloidogyne hapla*) is among the economically significant and widely distributed temperate nematodes with morphologically and genetically indistinguishable populations that show parasitic variability (PV) in the same plant host [1]. Parasitic variability is measured by how *M. hapla* populations induce galling and reproduce over a given period after inoculation. This nematode occurs across mineral and muck soils characterized by varying degradations and diverse microbial communities [1]. In general, a myriad of soil microbes have a varying association with *M. hapla* and other nematodes [2,3,4,5,6]. These associations range from fungal species and diverse bacteria that may attach to and parasitize the cuticle of *M. hapla* [4,5] to lethal-parasitism such as by *Pasteuria* sp. [2], to those microbes that may diminish [3] or enhance *M. hapla*’s ability to infect host plants [6]. Also, associations of *Paraburkolderia* (bacteria) and *Lectera* or *Penicillium* (fungi) with the cuticle of *M. hapla*, *M. incognita*, and *Pratylenchus penetrans* have been shown [3,4]. However, little is known about whether or not any associations exist between *M. hapla* distribution and PV and soil microbiome. Moreover, a lack of information on *M. hapla* distribution and PV relative to the soil biophysicochemical and soil health conditions is among the major knowledge gaps in understanding the cause-and-effect relationships of nematode PV.

One way to addressing the knowledge gaps is to establish the soil health conditions as described by the Ferris et al. [7] soil food web (SFW) model. The SFW model applies biological, ecological, and mathematical principles to changes in the beneficial nematode population density as a function of reproduction and food resources (enrichment index), and life history and resistance to disturbance (structure index) to distinguish the nutrient cycling potential and agroecosystem fitness as follows [7]: (a) disturbed (enriched and unstructured), (b) maturing (enriched and structured, best case), (c) depleted (resource-limited and structured), or (d) degraded (resource-limited and minimally structured, worst case). Recent studies analyzed soil where *M. hapla* exists in the lower peninsula of Michigan (USA) and the PV of populations therein and revealed three key points [8,9,10,11]. Firstly, *M. hapla* was distributed in mineral and muck soils with disturbed and/or degraded soil health conditions [8,9]. Secondly, *M. hapla* populations isolated from mineral soils with degraded soil health conditions had a higher PV than populations from similar muck soils and those from mineral and muck soils with disturbed conditions [10]. Thirdly, an analysis of the soil microbiome in the soils where *M. hapla* populations were isolated from identified 39 bacterial and 44 fungal core-microbiome members, defined by the abundance and incidence of taxa across fields [11], while 1065 indicator bacteria were associated with soil health conditions and/or *M. hapla* occurrence [1]. Indicator species are defined as those taxa having an increased occurrence or abundance associated with a specific condition or trait, such as PV [12]. However, it remains an open question whether and how core or indicator microbiome taxa are associated with PV in *M. hapla*.

Any positive or negative association between *M. hapla* populations and soil microbes is likely to start in the environment in which they co-exist and when the nematode’s life stages are exposed to the soil. Eggs and second-stage juveniles are two of the five *M. hapla* life stages that have direct exposure to the environment outside the root [13]. Eggs are laid in a gelatinous matrix inside the root or protruding out of the root. As the second-stage juvenile hatches from the egg and migrates through the soil to find the host roots, it has the most direct exposure to soil microbes within the environment they exit. What type(s), if any, of association(s) occur between the soil microbes and *M. hapla* populations showing PV as a function of the second-stage juveniles’ exposure to the soil environment is unknown. In this context, we define association as the presence or absence of microbes with the *M. hapla* population.

This study is a part of a project whose goal is to identify the mechanisms of *M. hapla* PV through understanding the environment in which *M. hapla* exists. The study presented herein expands the base line biophysicochemical information of soil environments showing *M. hapla* PV [8] by focusing on associations, or the lack of, between *M. hapla* populations showing PV and their microbiome. Our objective was to characterize the composition and function of bacterial communities associated with *M. hapla* populations isolated from mineral and muck soil fields with disturbed and degraded SFW conditions and greenhouse-raised sub-populations showing PV [8]. We know that these *M. hapla* populations have PV [10] and that there are core bacterial- and fungal-microbiomes as well as indicator bacteria associated with soil health conditions and/or *M. hapla* occurrence [1]. We hypothesize that the presence and/or absence of specific bacteria or functional groups is associated with *M. hapla* PV. Knowing whether or not there is an association between specific bacteria and *M. hapla* populations will advance our understanding of the environment in which *M. hapla* exists and potentially lead towards identifying cause-and-effect relationships of its PV.

## 2. Materials and Methods

### 2.1. Origin of Meloidogyne hapla Populations

**Figure 1:**
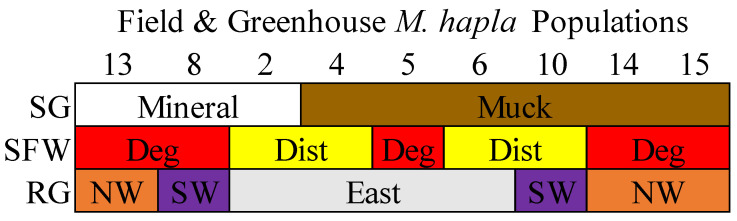
A description of the nine *M. hapla* populations isolated from the field and greenhouse populations of different soil groups (SG: mineral [white] and muck [brown]), soil food web conditions (SFW: Deg—degraded [red], Dist—disturbed [yellow]) and regions (RG: east [grey], SW—southwest [purple] and NW—northwest [orange]). The sequence of *M. hapla* populations from mineral soils are arranged from high to low parasitic variability (PV) but the muck populations with low PV (reproductive potential) are arranged numerically. See the corresponding map from where the nine *M. hapla* populations were sourced in Figure S1.

Three *M. hapla* populations (2, 8, and 13) from mineral soils and six populations (4, 5, 6, 10, 14, and 15) from muck soils were collected from selected vegetable production fields in the eastern, southwestern, and northwestern regions in the lower peninsula of Michigan, USA [8]. Populations 13, 14, and 15 were from the northwest, Populations 8 and 10 from the southwest, and Populations 2, 4, 5, and 6 were from the eastern regions (Figure S1). Populations 5, 8, 13, 14, and 15 came from degraded soil health conditions and Populations 2, 3, 6, and 10 from disturbed soil health conditions (Figure 1 [10] and Figure S1). When tested on tomato cv Rutgers under greenhouse conditions, Population 13 had the highest reproductive potential compared to all of the other populations, and the reproductive potential of Population 8 was greater than that of Populations 2, 4, 5, 6, 10, 14, and 15 [10].

### 2.2. The Greenhouse Maintenance of Meloidogyne hapla Populations

In this study, nine field and nine greenhouse *M. hapla* populations were used. The field populations were isolated from the original soil samples. The greenhouse populations were isolated from single egg mass cultures [14] maintained on tomato (*Solanum lycopersicum*) cv. ‘Rutgers’ over three years in a steam-sterilized (100 °C for 8 h) mix of top soil, sphagnum peat, and sand (supplied by the Michigan State University Plant Science Greenhouses [MSUPSG], East Lansing, MI, USA) [5]. Briefly, an egg mass was randomly selected from each of the populations, carefully removed from the roots of the original culture using clean tweezers and inoculated into the root zone of two-week-old tomato seedlings contained in a 400 cm^3^ clay pot with MSUPSG soil. The cultures were maintained in a greenhouse with controlled conditions: a temperature of 28 ± 3 °C, a 16 h day and 21 ± 3 °C, and an 8 h dark period. The plants were watered as required on a daily basis and fertilized twice weekly with Scotts’ Professional 20-20-20 (N-P-K) commercial mix (Marysville, OH, USA).

### 2.3. The Sampling of Field and Greenhouse Meloidogyne hapla Populations and DNA Extraction

Both the field and greenhouse *M. hapla* populations were extracted from a sub-sample of 100 cm^3^ of the soil using a semi-automatic elutriator (custom built, East Lansing, MI, USA), following the procedure outlined by Melakeberhan et al. [15] and Avendaño et al. [16]. Briefly, a mixture of a 1:1:3 ratio of soil, dish soap (non-phosphate, Cascade [Procter & Gamble, St Louis, MO, USA]), and tap water passed through the elutriator, filtered through sieves of decreasing sizes (850 µm, 250 µm, and 20 µm). A solution of 456 g sugar per L of tap water was added to the sediment and centrifuged at 4000 rpm for 4 min. The resulting suspension was poured through a clean 20 µm sieve, and the contents of the sieve were carefully rinsed with sterile distilled water and placed in sterile Petri dishes. Five second-stage juveniles from each population were individually picked with a sterile needle (Genesse Scientific, Morrisville, NC, USA, model 59-AWP-B) under a stereo microscope (Zeiss, Oberkochen, Germany, model AX10) and each placed into a 30 µL worm lysis mixture composed of 950 µL worm lysis solution (see Table S1) and 50 µL Proteinase K (Thermo Fisher Scientific, Waltham, MA, USA) for DNA extraction. Each of the five individual nematode in the lysis mixture underwent cycles of freezing at −80 °C for 10 min, thawing at 25 °C, and then an incubation at 60 °C for 60 min followed by 95 °C for 15 min in a thermocycler. The resulting DNA product representing all parts of the entire nematode was then stored at −80 °C.

### 2.4. Library Preparation

Amplicon libraries for Illumina MiSeq were prepared targeting the V4 region of the 16S bacterial rDNA using the 515F and 806R primers [17]. The library construction followed a three-step PCR protocol detailed in Table S2 [17,18]. Briefly, unmodified primers were used to enrich the target taxa in the first step, followed by frameshift incorporating primers [19,20] in the second step. Subsequently, a 10-nucleotide indexing set of barcodes and Illumina adapters were incorporated prior to sequencing [19,20]. To prevent the amplification of the mitochondria and chloroplast, PNA blocking clamps were employed [21]. Gel electrophoresis confirmed the amplification of PCR products, which were then normalized to concentrations of 1–2 ng/µL using the SequalPrep Normalization Plate Kit. After normalization, the samples were pooled and concentrated using Amicon Ultra 0.5 mL 50 K filters, followed by cleaning with Agencourt AMPure XP magnetic beads to remove primer dimers and small fragments. The cleaned libraries were sequenced using an Illumina Miseq V3 600 cycles kit at the Michigan State University Genomics Core. Sequenced samples were demultiplexed using Sabre software (https://github.com/najoshi/sabre accessed on 15 January 2022) [22], and the sequences are available in the Sequence Read Archive (SRA) of the National Center for Biotechnology Information (NCBI) under BioProject PRJNA854890.

### 2.5. Sequencing

Bioinformatic analyses of the 16S rDNA sequences were conducted in Qiime 2 version 2019.1 [23]. An initial quality assessment was performed using FastQC (http://www.bioinformatics.babraham.ac.uk/projects/fastqc/ accessed on 15 January 2022), and only forward reads were considered for the subsequent analysis. The quality distributions of the 16S library were analyzed using the q2-quality-filter plugin. Error modeling, de-replication, and denoising were performed using the default values of the q2-deblur plugin. Additionally, primers were trimmed, and sequences were truncated to 200 nucleotides. Chimera removal was performed using the q2-vsearch plugin, which implements the UCHIME algorithm to identify and filter out chimeric sequences. De novo clustering of the feature table was performed to group sequences into operational taxonomic units (OTUs) at 97% identity, following the standard practice for defining OTUs in microbiome studies [23]. OTUs with fewer than 5 reads were removed to ensure the robustness of downstream analyses. A taxonomy assignment of the representative 16S sequences was carried out using the ACT tool (SINA version 1.2.12—https://www.arb-silva.de/aligner/ accessed on 20 January 2022) of the SILVA online database (version 138, https://www.arb-silva.de/documentation/release-138/ accessed on 20 January 2022) [24], with specific settings for minimum identity and reject sequences. The classification of the sequences was based on the SILVA database. All analyses are accessible on GitHub (desktop version 3.4.7) at https://github.com/larteyis/PAPER-Bacterial-composition-diversity-and-functional-groups-associated-with-Meloidogyne-hapla-popul (accessed on 21 March 2023).

### 2.6. Statistical and Data Analyses

Data files containing OTU tables, taxonomy, mapping, and OTU sequences were loaded into the R (version 4.0.2) statistical environment [25] and used to create a phyloseq object for further analysis, using the phyloseq package (version 1.34.0) [26]. OTU contaminants were removed with a negative control using the decontam package (version 1.10.0) [27]. Afterwards, a sample summary and three sets of analyses were performed.

Firstly, the bacterial phyla and genera composition, and relative abundance were visualized with stacked bar plots for field and greenhouse populations. The core microbiome, defined as the set of microbes consistently present across populations, was assessed in three distinct groups: (a) all field populations, (b) all greenhouse populations, and (c) a combined group consisting of both field and greenhouse populations. Afterward, the alpha diversity was estimated for observed richness [28] and the Shannon diversity [29] with the microbiome (version 1.12.0) and vegan (version 2.5-7) packages [30], and boxplots of the alpha diversity data created with the ggplot2 package (version 3.3.5) [31]. In order to determine statistical differences, the Kruskal–Wallis test in the stats package was conducted across the populations [25].

Next, we visualized patterns of the compared microbial communities by soil group and SFW conditions. Firstly, OTUs of less than 5 reads in a sample were filtered out to account for PCR errors and artifacts [32]. Next, the metagenomeseq package (version 1.32.0) [33] was used to normalize the data by cumulative sum scaling. The soil group and SFW microbial community patterns associated with *M. hapla* populations were investigated by creating principal coordinates analysis (PCoA) plots with the ordinate and plot_ordination functions. The statistical significance of the microbial community patterns was tested with PERMANOVA as implemented by the adonis function of the vegan package (version 2.5-7). Afterwards, the within-sample variance (homogeneity of variance) were statistically tested with the “betadisper” function found in vegan.

Thirdly, functional groups of the microbial genera were assigned based on known metabolic and ecological roles as reported in the scientific literature. The functional categories were animal-pathogenic, anti_bacteria, anti-fungi, antibiotic-resistant, enhanced-nematode-parasitism, iron reducing, nematicidal, nitrogen_fixer, others, plant_growth_promoter, plant_pathogenic, polysaccharide-degrader, root_knot_nematode_associated, soybean-cyst-associated, and suppressive_soil bacteria. In cases where little or no information existed, we classified such genera as other. Across field and greenhouse *M. hapla* populations, the functional groups were visualized through stacked bar plots. All the analyses performed are accessible on github, provided in the link above.

## 3. Results

### 3.1. Summary of the Samples Analyzed

In this study, 99,828 sequences were initially obtained from 90 samples representing five samples for each of the nine field and nine greenhouse *M. hapla* populations. Following quality control and filtering procedures, which included the removal of low-quality sequences, chimeric sequences, and non-target organisms, 63,081 rDNA sequences were retained for analysis. An average of 971.42 sequences, ranging between 5 and 23,402 sequences per sample, were retained. These filtered sequences were used to characterize the microbiome associated with each nematode sample. The final dataset formed the basis of the subsequent analyses of microbial diversity and composition across the field and greenhouse populations.

### 3.2. Alpha Diversity

**Figure 2:**
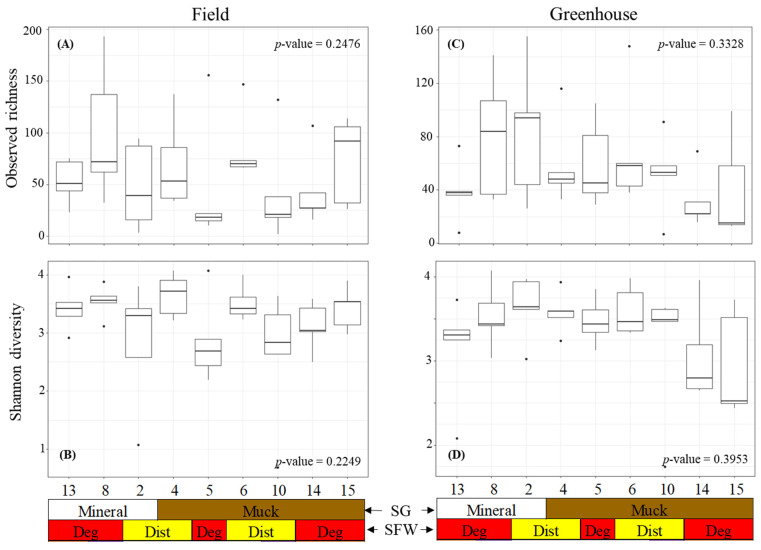
Alpha diversity boxplots showing the bacterial (**A**) field observed richness, (**B**) field Shannon diversity, (**C**) greenhouse observed diversity, and (**D**) greenhouse Shannon diversity of the *M. hapla* populations originating from different soil groups (SG: mineral [white] and muck [brown]) and soil food web conditions (SFW: Deg—degraded [red], Dist—disturbed [yellow]). Outliers on boxplots are displayed as dots. Kruskal–Wallis tests were performed to determine significant differences across the fields and *p*-values shown. Each numbered boxplot represents an *M. hapla* population in the field or the greenhouse.

The alpha diversity was measured using the observed richness and Shannon diversity of the bacterial communities associated with the field and greenhouse *M. hapla* populations (Figure 2). The observed richness and Shannon diversity were similar across the field populations (Figure 2A). Neither the soil group nor SFW conditions associated with the populations significantly influenced the richness and Shannon diversity. Similarly, the greenhouse populations had a similar observed richness and Shannon diversity regardless of the soil groups or SFW conditions previously associated with populations (Figure 2B).

### 3.3. Beta Diversity

**Figure 3:**
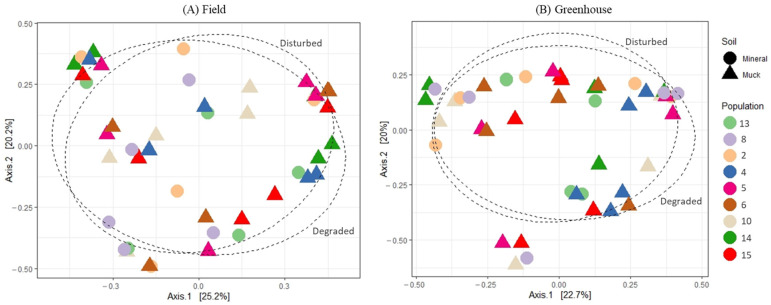
Principal coordinates analysis plots, based on Bray–Curtis dissimilarity, of the bacterial communities associated with field (**A**) and greenhouse (**B**) *M. hapla* populations originating from different soil groups (muck—triangle, and mineral—circle) and soil food web conditions (degraded and disturbed). Soil food web categories were separated with a 70% ellipse.

The principal coordinate analysis (PCoA) of the bacterial communities associated with either field or greenhouse *M. hapla* populations did not reveal any distinct patterns relative to the soil groups or SFW conditions from where the soil samples were collected (Figure 3A,B). Similarly, neither soil groups, SFW conditions, or their interactions had a significantly different PERMANOVA (*p* > 0.05; perm. = 9999), and variance tests showed no significant differences between groups (Table 1). However, a total of a 45.4% variance (axis1: 25.2%, and axis2: 20.2%) and 42.7% variance (axis1: 22.7%, and axis2: 20.0%) could be explained by the graphs of field and greenhouse microbial communities, respectively (Figure 3A,B).

### 3.4. Community Composition

**Figure 4:**
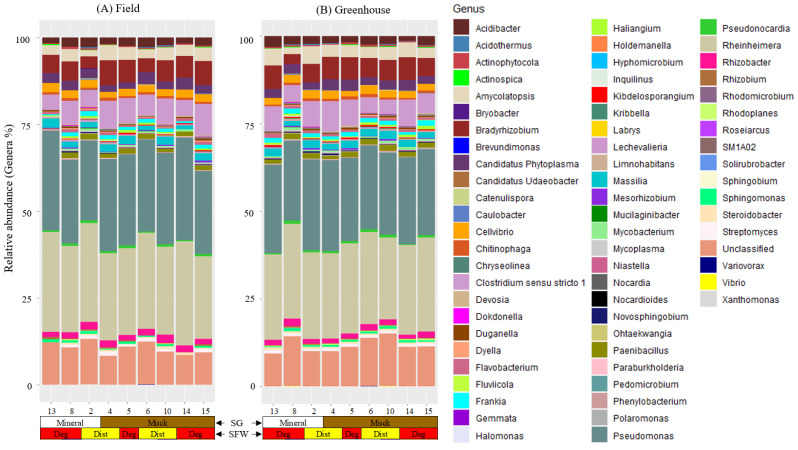
Stacked bar plots showing the relative abundance of bacterial genera associated with (**A**) field and (**B**) greenhouse *M. hapla* populations originating from different soil groups (SG: muck [brown] and mineral [white]) and soil food web conditions (SFW: Deg—degraded [red] and Dist—disturbed [yellow]). Sixty genera were detected in both the field and greenhouse populations, five (*Norcardia*, *Rhodomicrobium*, *Gemmata*, *Fluviicola*, and *Vibrio*) in only field populations, and one (*Steroidobacter*) in only greenhouse populations. Other OTUs which were not assigned genera were labelled as unclassified. Colors of the bacteria and fungi correspond with colors in the stacked bar plots and each bar represents a field. The relative abundance of bacterial genera was variable across the field and the greenhouse populations. Sequences were assigned to taxonomic groups using the ACT (alignment, classification, tree service; version 1.2.12; https://www.arb-silva.de/aligner/ accessed on 20 January 2022) tool of SILVA (version 138) online database. Each numbered vertical bar of the plot represents an *M. hapla* population in the field or the greenhouse.

We detected nine bacterial phyla and 65 genera in the field populations and 61 genera in the greenhouse populations of *M. hapla* populations (Figure S2 and Figure 4). The phyla Actinobacteria, Bacteroidetes, Firmicutes, Myxococcota, and Proteobacteria were present in all of the field and greenhouse populations; whereas, the presence of Acidobacteria, Chloroflexi, Planctomycetes, and Verrucomicrobia had a low and variable relative abundance across these two sets of populations (Figure S2A,B).

We found a high variability in the presence/absence and relative abundance of the bacterial communities associated with the populations from mineral or muck soils with either disturbed or degraded SFW conditions, or a high, medium, or low PV category (Figure 4). *Acidibacter*, *Actinophytocola*, *Amycolatopsis*, *Bradyrhizobium*, *Candidatus Phytoplasma*, *Cellvibrio*, *Chitinophaga*, *Clostridium sensu stricto 1*, *Duganella*, *Flavobacterium*, *Frankia*, *Lechevalieria*, *Massilia*, *Paenibacillus*, *Pseudomonas*, *Pseudonocardia*, *Rheinheimera*, and *Rhizobacter* were among the core microbiota genera shared across both the field and greenhouse populations of *M. hapla* (Figure 4A,B). No unique core microbes were identified within the greenhouse populations alone. In addition to those core genera shared across both environments, *Dongia*, *Haliangium*, *Kibdelosporangium*, *Mycobacterium*, *Novosphingobium*, *Rhizobium*, *Rhodoplanes*, *Sphingomonas*, *Steroidobacter*, and *Streptomyces* were unique to the field populations (Figure 4A,B).

Population 13 had *Inquilinus* in common with Populations 5 and 8 and *Caulobacter* with Populations 5 and 15 in the field, and *Halomonas* with Populations 8 and 10 and *Limnohabitans* with Populations 2, 4, 5, and 15 in the greenhouse. *Mesorhizobium* and *Ohtaekwangia* in the field and *Devosia*, *Kibdelosporangium*, *Mycobacterium*, and *Steroidobacter* in the greenhouse were absent in Population 13. *Streptomyces* in the greenhouse and *Acidothermus*, *Devosia*, *Limnohabitans*, and *Mycoplasma* in the field were absent in Population 13 and one or more of the low PV category populations (Figure 4A,B).

*Brevundimonas*, *Candidatus Udaeobacter*, *Hyphomicrobium*, *Mucilaginibacter*, *Nocardioides*, *Roseiarcus*, and *Solirubrobacter* in the field and *Hyphomicrobium* in the greenhouse was limited to Population 8. The Population 8 microbiome included *Catenulispora*, *Gemmata*, *Pedomicrobium*, *Rhodomicrobium*, *Sphingobium*, *Phenylobacterium*, *Variovorax*, and *Xanthomonas* in the field, *Brevundimonas*, *Chryseolinea*, *Kribbella*, *Mucilaginibacter*, *Mycoplasma*, *Niastella*, *Novosphingobium*, *Ohtaekwangia*, *Paraburkholderia*, and *Rhodoplanes* in the greenhouse, and *Dokdonella*, *Labrys*, *Nocardia*, and *Polaromonas* in both the field and greenhouse populations in common with one or more of the low PV category populations (Figure 4A,B).

The low PV category populations had some unique taxa that were present or absent as well. The presence of *Chryseolinea* and *Fluviicola* in the field, *Holdemanella*, *Nocardioides*, *Pedomicrobium*, *Sphingobium*, *Solirubrobacter*, *SM1A02*, and *Xanthomonas* in the greenhouse, *Bryobacter* in both the field and greenhouse populations were limited to Populations 2. Similarly, *Holdemanella* and *SM1A02* in the field and *Roseiarcus* in the greenhouse were specific to Population 15 and 6, respectively. *Actinospica* in Populations 2, 4, and 14, and *Kribbella* in Populations 5 and 15 were absent in the field (Figure 4A).

### 3.5. Functional Groups

**Figure 5:**
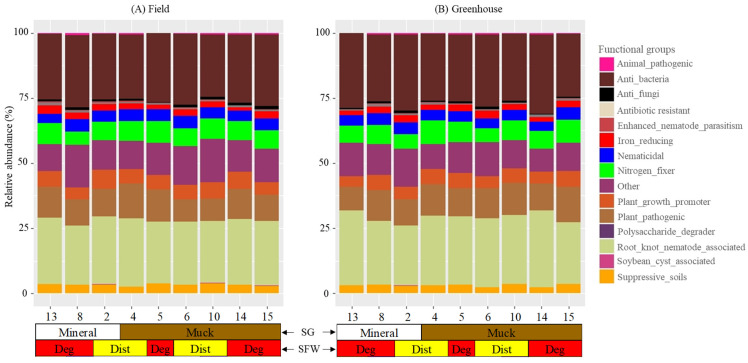
Stacked bar plots showing the relative abundance of the bacterial functional groups associated with field (**A**) and greenhouse (**B**) *M. hapla* populations originating from different soil groups (SG: mineral [white] and muck [brown]) and soil food web conditions (SFW: Deg—degraded [red], Dist—disturbed [yellow]). The greenhouse samples were isolated from tomato roots growing in a sterilized soil media for multiple generations. A list of bacterial genera assigned to each of the 15 functional groups is presented in Table 2. All of the genera which did not have a known function were grouped as “other”. Each numbered vertical bar of the plot represents an *M. hapla* population in the field or the greenhouse.

Sixty-five of the bacterial genera detected in the field and greenhouse *M. hapla* populations belonged to 14 functional bacterial groups (Figure 5; Table 2). The functional groups and the numbers of genera (in brackets) were as follows: animal-pathogenic (2), anti_bacteria (3), anti-fungi (4), antibiotic-resistant (1), enhanced-nematode-parasitism (1), iron reducing (1), nematicidal (12), nitrogen_fixer (5), others (12), plant_growth_promoter (8), plant_pathogenic (5), polysaccharide-degrader (1), root_knot_nematode_associated (2), soybean-cyst-associated (1), and suppressive_soil bacteria (8) (Table 2). Another 12 identified genera (*Bryobacter*, *Catenulispora*, *Dokdonella*, *Fluviicola*, *Holdemanella*, *Hyphomicrobium*, *Inquilinus*, *Labrys*, *Limnohabitans*, *Pseudonocardia*, *Roseiarcus*, and *SM1A02*) with little known functions were classified as other. The numbers of the bacterial functional groups and genera had a varying relative abundance with no clear trends between the field and greenhouse *M. hapla* populations (Figure 5; Table 2). The functional groups and genera are described as common to both sets of populations, varying, or in a specific presence or absence in certain field and greenhouse populations.

Animal_pathogenic (*Flavobacterium*), anti-bacteria (*Lechevalieria* and *Rheinheimera*), nematicidal (*Cellvibrio*, *Chitinophaga*, and *Duganella*), iron_reducing (*Acidibacter*), nitrogen-fixer (*Bradyrhizobium* and *Frankia*), other (*Pseudonocardia*), plant-growth-promoter (*Amycolatopsis* and *Paenibacillus*), plant-pathogenic (*Candidatus* Phytoplasma, *Clostridium sensu stricto 1*, and *Rhizobacter*), root-knot nematode associated (*Pseudomonas*) and suppressive soil (*Actinophytocola* and *Massilia*) functional groups were present in the field and greenhouse populations (Figure 5A,B). Animal-pathogenic (*Mycobacterium*), anti-bacteria (*Kibdelosporangium*), anti_fungi (*Acidothermus*, *Chryseolinea*, *Haliangium*, and *Paraburkholderia*) and enhanced_nematode_parasitism (*Novosphingobium*), nematicidal (*Streptomyces*), nitrogen_fixer (*Rhizobium*), plant_growth_promoter (*Sphingomonas*), and root_knot_nematode_associated (*Rhodoplanes*) were present in all of the field populations but variably in the greenhouse populations. The presence or absence of the antibiotic-resistant (*Candidatus Udaeobacter*), polysaccharide-degrader (*Sphingobium*) and the rest of the genera in the other 12 functional groups varied in both the field and greenhouse populations (Figure 5A,B).

Population 13 had in common the presence of *Inquilinus* (other) with Populations 5 and 8 and plant_growth_promoters *Caulobacter* with Populations 5 and 15 in the field, and *Halomonas* with Populations 8 and 10 and *Limnohabitans* (other) with Populations 2, 4, 5, and 15 in the greenhouse. Population 13 had the absence of *Acidothermus* (anti-fungi), and *Mycoplasma* (plant_pathogenic) in the field and *Streptomyces* (nematicidal) in the greenhouse and *Devosia* (nematicidal) in the field and greenhouse in common with one or more of the low PV category populations. The absence of *Mesorhizobium* (nitrogen_fixer) and *Ohtaekwangia* (suppressive soil bacteria) in the field, and *Devosia* (nematicidal) *Kibdelosporangium* (anti-bacteria), *Mycobacterium* (animal pathogenic) and *Steroidobacter* (suppressive_soil bacteria) in the greenhouse was limited only to Population 13 (Figure 5A,B). The presence of *Candidatus Udaeobacter* (antibiotic-resistant) and *Brevundimonas* and *Mucilaginibacter* (plant growth promoters), *Roseiarcus* (other), and *Nocardioides* and *Solirubrobacter* (nematicidal) in the field and *Hyphomycrobium* (other) in both the field and greenhouse was limited to Population 8. Population 8 had plant growth promoters *Brevundimonas* and *Mucilaginibacter Niastella*, *Ohtaekwangia*, and *Kribbella* (suppressive_soil bacteria), *Paraburkholderia* (anti fungi), *Mycoplasma* (plant pathogenic), *Chryseolinea* (anti-fungi), *Novosphingobium* (enhanced-nematode-parasitism), and *Rhodoplanes* (root_knot_nematode_associated) in the greenhouse, and *Catenulispora* (other), *Gemmata*, *Pedomicrobium*, *Phenylobacterium*, *Xanthomonas* (nematicidal), *Rhodomicrobium* (nitrogen_fixer), *Sphingobium* (polysaccharide-degrader) and *Variovorax* (suppressive_soil bacteria) in the field, and *Dokdonella* and *Labrys* (others), *Nocardia* (plant_growth_promoters), and *Polaromonas* (soybean-cyst-associated) in the greenhouse and field in common with one or more of the low PV category *M. hapla* populations (Figure 5A,B).

The presence of Chryseolinea (anti-fungi) and Fluviicola (others) in the field, Holdemanella and SM1A02 (others), Nocardioides, Pedomicrobium, Solirubrobacter, and Xanthomonas (nematicidal), and Sphingobium (polysaccharide-degrader) in the greenhouse, Bryobacter (others) in both the field and greenhouse populations were limited to Populations 2. Similarly, Holdemanella and SM1A02 (others) in the field and Roseiarcus (other) in the greenhouse were specific to Population 15 and 6, respectively. Suppressive soil bacteria Actinospica in Populations 2, 4, and 14, and Kribbella in Populations 5 and 15 were absent in the field (Figure 5A,B).

### 3.6. Bacteria in M. hapla Populations and in Core and Indicator Groups in Field Soils

We previously reported the presence of 39 core bacteria OTUs belonging to 11 identified genera in soils across all of the fields where the *M. hapla* populations were isolated from (Table S3) and 25 indicator OTUs associated with the absence (OTU 1–OTU 16) and presence (OTU 17–OTU 25) of *M. hapla* (Table S4) [1]. Out of the 11 core bacterial genera, two were detected within *M. hapla* populations. These included *Paenibacillus* (plant_growth_promoter) in all of the field and greenhouse populations, and *Rhodoplanes* in all of the field populations, but they were conspicuously absent from greenhouse populations.

The core bacteria presence limited to one or specific *M. hapla* populations were *Sphingobium* (polysaccharide-degrader) in field Population 2; *Devosia* (nematicidal) in field Populations 10 and 13, and all of the greenhouse populations except in Population 13; and *Rhodoplanes* (root_knot_nematode_associated) in all of the field populations, and greenhouse Populations 2, 5, 10, 4, and 8 (Table S3).

Only two of the 25 bacterial indicator OTUs associated with the absence (OTU 1–OTU 16) or presence (OTU 17–OTU 25) of *M. hapla* were detected in the nematodes. These included the presence of *Rhizobium* sp. (OTU 6; nitrogen fixer) in all of the field *M. hapla* populations, and all of the greenhouse populations with the exception of greenhouse Population 14; and *Brevundimonas* sp. (OTU 9; plant_growth_promoter) in field and greenhouse Population 8, and in greenhouse Population 2 (Table S4).

## 4. Discussion

This study aimed to enhance our understanding of the relationships between *M. hapla* populations with high- (Population 13), medium- (Population 8), and low-PV (Populations 2, 4, 5, 6, 10, 14, and 15 [10]) and the microbiome of *M. hapla*. Populations 2, 8, and 13 are from mineral soil and the rest are from muck soil. After establishing bacterial and fungal core-microbiomes and bacterial OTUs associated with either soil health conditions and/or *M. hapla* occurrence in the field [1], we examined the composition and function of bacteria associated with field and greenhouse *M. hapla* populations. Our results improve the understanding of the soil conditions in which *M. hapla* PV exists by describing bacterial communities and functional groups common to both the field and the greenhouse populations, and variations relative to the high-, medium- and low-PV of the nematode populations.

### 4.1. Community, Diversity, and Composition

The richness- and Shannon-based analyses showed no difference in the diversity of bacterial communities between field and greenhouse *M. hapla* populations. Similarly, the PCoA showed no clear pattern by population, soil group, or soil food web conditions, which indicates that the bacterial diversity is independent of the soil and SFW conditions [34]. While the overall diversity of the bacterial communities in the *M. hapla* microbiome was similar, there were differences in the abundance and composition of genera in the field and greenhouse populations.

The bacterial phyla and genera detected had varying proportions with no clear trend between the field and greenhouse *M. hapla* populations. The phyla Actinobacteria, Bacteroidetes, Firmicutes, Myxococcota, and Proteobacteria and the genera *Acidibacter*, *Actinophytocola*, *Amycolatopsis*, *Bradyrhizobium*, *Candidatus Phytoplasma*, *Cellvibrio*, *Chitinophaga*, *Clostridium sensu stricto*, *Duganella*, *Flavobacterium*, *Frankia*, *Lechevalieria*, *Massilia*, *Paenibacillus*, *Pseudomonas*, *Pseudonocardia*, *Rheinheimera*, *Rhizobacter*, and the unclassified genera were common to all of the field and greenhouse *M. hapla* populations and at high proportions. Actinobacteria, Bacteroidetes, Firmicutes, Proteobacteria, and Chloroflexi are known to be associated with *M. hapla* suppression [6] and *Verrucomicrobium* synthesizing amino acids in the nematode *Xiphenema americanum* as a nutritional mutualist [35]. Little is known about the role of Myxococcota and Planctomycetes relative to nematodes. While there is little published association of these phyla and/or genera and their varying proportions on *M. hapla* PV, it can be assumed that these bacterial communities may have the same effect on both sets of the populations [3]. On the other hand, the presence of the phyla Acidobacteria, Chloroflexi, Planctomycetes, and Verrucomicrobia and the genera *Haliangium*, *Kibdelosporangium*, *Mycobacterium*, *Novosphingobium*, *Rhizobium*, *Rhodoplanes*, *Sphingomonas*, and *Streptomyces* in all of the field, but variably in the greenhouse populations shows differences between the two sets of *M. hapla* populations. Whether or not the variable presence of these bacterial communities may be related to the observed PV differences among the populations [10] remains to be determined.

Interestingly, the presence of bacterial communities between the two sets of *M. hapla* populations differed among the high (Population 13), medium (Population 8), and low PV category (Populations 2, 4, 5, 6, 10, 14, and 15) populations. For example, Population 13 only had the presence of *Inquilinus* in the field and *Halomonas* in the greenhouse in common with Population 8 and/or Populations 5 and 10, and *Caulobacter* and *Limnohabitans* in the greenhouse with two or more of the low PV category populations. Population 8, on the other hand, had *Catenulispora*, *Gemmata*, *Pedomicrobium*, *Rhodomicrobium*, *Sphingobium*, *Phenylobacterium*, *Variovorax*, and *Xanthomonas* in the field, *Brevundimonas*, *Chryseolinea*, *Kribbella*, *Mucilaginibacter*, *Mycoplasma*, *Niastella*, *Novosphingobium*, *Ohtaekwangia*, *Paraburkholderia*, and *Rhodoplanes* in the greenhouse, and *Dokdonella*, *Labrys*, *Nocardia*, and *Polaromonas* in both the field and greenhouse populations in common with one or more of the low PV category populations. This shows that the medium and low PV category *M. hapla* populations have more bacterial compositions associated with them in common than with Population 13.

Population 13 did not have bacteria genera uniquely present with it as Populations 8 and several of the low PV category populations did. These included the presence of *Brevundimonas*, *Candidatus Udaeobacter*, *Hyphomicrobium*, *Mucilaginibacter*, *Nocardioides*, *Roseiarcus*, and *Solirubrobacter* in the field, and *Hyphomicrobium* in the greenhouse was limited to Population 8. *Chryseolinea* and *Fluviicola* in the field, *Holdemanella*, *Nocardioides*, *Pedomicrobium*, *Sphingobium*, *Solirubrobacter*, *SM1A02*, and *Xanthomonas,* and *Bryobacter* in the greenhouse were limited to Population 2. While the absence of *Streptomyces* in the greenhouse and *Acidothermus*, *Devosia*, *Limnohabitans*, and *Mycoplasma* in the field were common to Population 13 and on or more of the low PV category populations, the absence of *Mesorhizobium* and *Ohtaekwangia* in the field and *Devosia*, *Kibdelosporangium*, *Mycobacterium*, and *Steroidobacter* in the greenhouse were unique to Population 13. If the absence of these bacterial genera in Population 13 have anything to do with its PV is yet to be determined. To better understand the potential associations of bacterial communities with *M. hapla* populations, however, it is worth considering the functions of the specific bacterial genera present in the nematodes.

### 4.2. Functional Groups and Their Habitats

Sixty-five of the identified bacterial genera in the nine phyla represented 14 known functional groups and 12 genera were of unknown (other) functions. The 18 genera commonly associated with all of the field and greenhouse *M. hapla* populations belonged to nine known and one unknown functional group(s). These were animal pathogens (*Flavobacterium*), anti-bacteria (*Lechevalieria* and *Rheinheimera*), nematicidal (*Cellvibrio*, *Chitinophaga* and *Duganella*), iron-reducing (*Acidibacter*), nitrogen-fixing (*Bradyrhizobium* and *Frankia*), plant-growth-promoting (*Amycolatopsis* and *Paenibacillus*), plant-pathogenic (*Candidatus Phytoplasma*, *Clostridium sensu stricto 1* and *Rhizobacter*), root-knot nematode associated (*Pseudomonas*), suppressive soil (*Actinophytocola* and *Massilia*), and other (*Pseudonocardia*) functional groups. *Flavobacterium* is a pathogen of the oriental beetle (*Blitopertha orientalis*). *Lechevalieria* produces rebeccamycin antibiotic and *Rheinheimera* toxins that kill *Euplotes aediculatus* [36,37]. *Cellvibrio*, *Chitinophaga*, *Duganella*, *Pseudomonas*, *Actinophytocola*, and *Massilia* are a part of a bacterial consortium that negatively impacts root-knot and cyst nematodes [38,39,40]. *Candidatus Phytoplasma* causes stunting and witches’ broom in several vegetable crops [41], *Clostridium sensu stricto 1* causes the soft rot disease of sweet potato [42], and *Rhizobacter* the gall disease of carrot [43]. *Amycolatopsis* enhances plant growth by inhibiting the charcoal rot disease caused by *Macrophomina phaseolina* [44] and *Paenibacillus* tomato growth and root-mass production infested with *M. incognita* [45]. *Acidibacter* is a mesophile that reduces iron [46] and *Bradyrhizobium* and *Frankia* are involved in plant root nodulation and nitrogen fixation [47,48]. However, little is known about the role of *Pseudonocardia* in soil.

The genera that were common to the field populations, but variable in the greenhouse populations, belonged to animal-pathogenic (*Mycobacterium*), anti-bacterial (*Kibdelosporangium*), anti-fungal (*Acidothermus*, *Chryseolinea*, *Haliangium* and *Paraburkholderia*), enhanced-nematode-parasitism (*Novosphingobium*), nematicidal (*Streptomyces*), nitrogen_fixer (*Rhizobium*), plant growth promoter (*Sphingomonas*), and root knot nematode associated (*Rhodoplanes*) functional groups. Given the diversity of the bacteria associated with the *M. hapla* populations, variations within functional groups are to be expected [49]. *Mycobacterium* causes tuberculosis in cattle and *Kibdelosporangium* produces antibiotic substances like cycloviracins, aricidins, and kibdelins [50,51,52,53]. *Acidothermus* suppresses the activity of arbuscular mycorrhizal fungi, *Chryseolinea* suppresses the *Fusarium* wilt of banana, *Haliangium* produces bioactive products against fungi, and *Paraburkholderia* suppresses the root rot fungal pathogen *Cylindrocarpon destructans* [54,55,56]. *Novosphingobium* synthesizes vitamin B12 which enhances *Pristionchus pacificus* parasitism against *Caenorhabditis elegans* [57] and *Streptomyces* with biofumigation is lethal to *M. incognita* [58]. *Rhizobium* fixes nitrogen in legumes for plant growth, *Sphingomonas* increases the lateral roots and root hairs of *Arabidopsis thaliana* [59,60] and *Rhodoplanes* was associated with *Meloidogyne* spp. [61]. However, if the variable presence of these genera in the greenhouse *M. hapla* populations is because of the difference between the field and greenhouse soils or other undescribed biological associations, or the lack of them, is unknown [62].

The medium- (Population 8) and low- (Populations 2, 4, 5, 6, 10, 14, and 15) PV populations had more bacterial genera in common than with the high PV, Population 13. For example, Population 8 alone had *Candidatus Udaeobacter* (antibiotic-resistant), *Nocardioides* and *Solirubrobacter* (nematicidal), *Brevundimonas* and *Mucilaginibacter* (plant growth promoters), and *Roseiarcus* and *Hyphomycrobium* (other functions). Moreover, the diversity of bacteria that Population 8 shared with the low PV category populations is particularly worth noting. These included *Chryseolinea* and *Paraburkholderia* (anti fungi), *Rhodoplanes* (root_knot_nematode_associated), *Novosphingobium* (enhanced-nematode-parasitism), *Kribbella* and *Variovorax* (suppressive_soil bacteria), *Polaromonas* (soybean-cyst-associated), *Brevundimonas* and *Mucilaginibacter Niastella*, *Nocardia* and *Ohtaekwangia* (plant_growth_promoters), *Gemmata*, *Pedomicrobium*, *Phenylobacterium*, *Xanthomonas,* and *Rhodomicrobium* (nitrogen_fixers), *Sphingobium* (polysaccharide-degrader), and *Catenulispora*, *Dokdonella*, and *Labrys* (other). *Candidatus Udaeobacter* thrives in environments concentrated with antibiotics [63]. *Nocardioides* has a negative relationship with *M. hapla* and *P. neglectus* numbers, and *Solirubrobacter* together with *Gemmata*, *Pedomicrobium*, *Phenylobacterium*, and *Xanthomonas* have a negative impact on *Meloidogyne* spp [40,64]. *Brevundimonas* increases the nitrogen intake to enhance potato growth, *Mucilaginibacter* promotes plant growth by enhancing rhizobacteria, *Rhodomicrobium* synthesizes enzymes that enable nitrogen fixation in plants, and *Nocardia* produces auxins that induce nodule-like structures to enhance the growth of *Casuarina glauca* [48,65,66,67]. *Kribbella*, *Niastella*, *Ohtaekwangia*, and *Variovorax* co-operatively suppress the plant parasitic nematode *Heterodera glycines* [39] and *Polaromonas* that was detected in a *Heterodera glycines* cyst [68]. *Sphingobium* produces enzymes which allows sugars to be degraded [69,70]. Not much is known about *Roseiarcus*, *Hyphomycrobium*, *Catenulispora*, *Dokdonella*, and *Labrys*. While how the presence of these genera may or may not be related to the populations with medium and low PV is yet to be determined, these data provide a basis for more targeted analyses of the associations, or the lack of, among these bacteria and *M. hapla* populations.

On the other hand, what Population 13 had in common with Population 8 and/or one or more of the low PV category populations were the presence of *Caulobacter* (plant_growth_promoter) and *Halomonas*, *Inquilinus*, and *Limnohabitans* (other or unknown functions) and the absence of *Acidothermus* (anti-fungi), *Mycoplasma* (plant_pathogenic), and *Devosia* and *Streptomyces* (nematicidal) in the populations from the field and/or greenhouse. *Caulobacter* increases root, leaf number, and leaf size in *Arabidopsis thaliana* [71] and *Mycoplasma* is a pathogen of plants like corn and citrus [72]. It is fair to assume that any effect of these bacteria on the high-, medium-, and low-PV populations may be similar. What separates Population 13 from the medium- and low-PV populations is the unique absence of *Mesorhizobium* (nitrogen_fixer) and *Ohtaekwangia* (suppressive soil bacteria) in the field, and *Kibdelosporangium* (anti-bacteria), *Steroidobacter* (suppressive_soil bacteria), *Devosia* (nematicidal), and *Mycobacterium* (animal pathogenic) in the greenhouse. *Steroidobacter* thrives in *Heterodera glycines* suppressive soils conditions with a relatively neutral pH; and *Devosia* in *M. hapla* suppressive soils decreasing galling and fecundity [39,73,74]. *Mesorhizobium* is commonly associated with the nodulation of legumes [75] and *Ohtaekwangia* is broadly adapted to aerobic, acidic, and alkaline conditions [76]. If and how the absence of the bacterial genera in Population 13 relates to its PV is unknown.

### 4.3. Connections with the Core and Indicator Groups Present in Field Soils

A total of 39 core and 25 indicator bacterial OTUs were identified across the fields including those from where the nematodes were isolated [1]. Core OTUs are defined as the most abundant and prevalent taxa across the fields [11], and indicator OTUs are characterized by an increased occurrence or abundance associated with a group of sites of similar conditions [12]. In soils collected from the fields from where the nine *M. hapla* populations were isolated, 39 core bacteria OTUs belonging to 11 known genera were identified [1]. The current study has identified some notable presence and/or absence of the four core (*Paenibacillus*, *Rhodoplanes*, *Devosia*, and *Sphingobium*) and two indicator (*Rhizobium* and *Brevundimonas*) bacteria from the field soil and bacteria present in the nine field and greenhouse *M. hapla* populations.

Out of the 11 core bacterial genera present in the soils from the fields (Table S3), *Paenibacillus* (plant_growth_promoter) was the only one present in all of the field and greenhouse *M. hapla* populations. *Rhodoplanes* (root_knot_nematode_associated) was present in all of the field populations and greenhouse Populations 2, 5, 10, 4 (low PV), and 8 (medium PV), but absent in the high-PV Population 13 and low-PV Populations 6, 14, and 15 (Table S3). *Devosia* (nematicidal) was present in field Populations 10 and 13 and in all of the greenhouse populations, but Population 13. *Sphingobium* (polysaccharide-degrader) was present only in *M. hapla* Population 2 from the field, suggesting a limited occurrence and/or association.

There were 25 bacterial indicator OTUs associated with the absence (OTU 1–OTU 16) or presence (OTU 17–OTU 25) of *M. hapla* in the soil from where the nematodes that were isolated were detected from (Table S4) [1], but only OTU 6 (*Rhizobium* sp.; nitrogen fixer) and OTU 9 (*Brevundimonas* sp.; plant_growth_promoter) were detected in this study. OTU 6 was present in all the field *M. hapla* populations and all of the greenhouse populations, but Population 14 (low PV), and OTU 9 in field and greenhouse Population 8, and in greenhouse Population 2 only. Thus, this suggests broad and narrow associations of the respective OTUs with the nematodes.

The fields where the nine *M. hapla* populations were isolated from had a wide range of soil texture and crops [8]; whereas, the greenhouse cultures of the nine populations were maintained in steam-sterilized soil (a mix of topsoil, sphagnum peat, and sand supplied by the Michigan State University Plant Science Greenhouses) [10]. If any, and how much, the differences in soil types and plant hosts may have contributed to the observed differences in associations among the core and indictor microbiomes in the soil and those detected in the nematodes is unknown. Moreover, what types of associations may or may not exist between the *M. hapla* populations and either the core and/or indicator OTUs is yet to be determined.

## 5. Conclusions

Understanding *M. hapla*‘s PV is difficult because of the complexities of the environments in which the interactions occur and the lack of information that connects and/or discounts any confounding factors therein. Building on previous studies that established connections among soil health conditions and *M. hapla* distribution and PV, and core and indicator microbiomes [8,9,10,11], this study furthers our understanding by determining the relationships between *M. hapla* field and greenhouse sub-populations with high- (Population 13), medium- (Population 8) and low-PV (Populations 2, 4, 5, 6, 10, 14, and 15) and bacterial communities in the nematodes. This study established key findings. Sixty-five genera in the field and 61 genera in the greenhouse population, with 12 genera of an unknown function and the rest belonging to 14 known functional groups were isolated from the nematodes. The bacterial richness and Shannon diversity did not significantly differ across field- and greenhouse-isolated *M. hapla* populations, yet the presence of bacterial genera and functional groups within *M. hapla* PV were variable. The medium- and low-PV *M. hapla* populations shared more bacterial compositions than either one shared with the high-PV population. The overlap and/or differences in the bacteria composition among and between the low-, medium-, and high-PV *M. hapla* populations provide a foundation for in-depth studies in establishing the potential cause-and-effect relationships of *M. hapla* PV.

## Figures and Tables

**Table 1:** Permutational multivariate analysis of the variance and multivariate homogeneity of groups dispersions analysis results of microbial communities associated with field and greenhouse *M. hapla* based on soil group (SG), region (RG), soil health (SFW) conditions, and interactions.

	Field *M. hapla*					Greenhouse *M. hapla*				
	PERMANOVA			DISPERSION		PERMANOVA			DISPERSION	
Variable	F-Value	R^2^	*p*-Value	F-Value	*p*-Value	F-Value	R^2^	*p*-Value	F-Value	*p*-Value
SG	1.101	0.025	0.347	2.23	0.627	0.472	0.011	0.879	0.008	0.922
RG	1.134	0.052	0.319	0.52	0.577	0.759	0.036	0.722	1.211	0.288
SFW	0.224	0.005	0.993	0.073	0.796	0.262	0.006	0.988	1.628	0.206
SG:SFW	0.775	0.054	0.755	0.095	0.963	0.662	0.046	0.885	0.727	0.529
SG:RG	0.859	0.099	0.697	0.221	0.942	0.517	0.062	0.994	0.497	0.78
SFW:RG	0.807	0.075	0.765	0.118	0.975	0.523	0.05	0.989	0.584	0.679
SG:RG:SFW	0.788	0.111	0.837	0.222	0.967	0.499	0.073	0.999	0.568	0.743

**Table 2:** The functional groups, phyla, and genera of bacteria found in the field and greenhouse *M.* hapla populations.

Functional Groups	Phyla	Genera
Animal_pathogenic	Actinobacteria	*Mycobacterium*
	Bacteroidetes	*Flavobacterium*
Anti_bacteria	Actinobacteria	*Kibdelosporangium*
	Actinobacteria	*Lechevalieria*
	Proteobacteria	*Rheinheimera*
Anti_fungi	Actinobacteria	*Acidothermus*
	Proteobacteria	*Paraburkholderia*
	Bacteroidetes	*Chryseolinea*
	Proteobacteria	*Haliangium*
Antibiotic_resistant	Verrucomicrobia	*Candidatus Udaeobacter*
Enhance_nematode_parasitism	Proteobacteria	*Novosphingobium*
Iron_reducing	Proteobacteria	*Acidibacter*
Nematicidal	Proteobacteria	*Cellvibrio*
	Bacteroidetes	*Chitinophaga*
	Proteobacteria	*Devosia*
	Proteobacteria	*Duganella*
	Planctomycetes	*Gemmata* *
	Actinobacteria	*Nocardioides*
	Proteobacteria	*Pedomicrobium*
	Proteobacteria	*Phenylobacterium*
	Actinobacteria	*Solirubrobacter*
	Actinobacteria	*Streptomyces*
	Proteobacteria	*Vibrio* *
	Proteobacteria	*Xanthomonas*
Nitrogen_fixer	Proteobacteria	*Rhizobium*
	Proteobacteria	*Bradyrhizobium*
	Actinobacteria	*Frankia*
	Proteobacteria	*Mesorhizobium*
	Proteobacteria	*Rhodomicrobium* *
Other	Acidobacteria	*Bryobacter*
	Actinobacteria	*Catenulispora*
	Proteobacteria	*Dokdonella*
	Bacteroidetes	*Fluviicola* *
	Firmicutes	*Holdemanella*
	Proteobacteria	*Hyphomicrobium*
	Proteobacteria	*Inquilinus*
	Proteobacteria	*Labrys*
	Proteobacteria	*Limnohabitans*
	Actinobacteria	*Pseudonocardia*
	Proteobacteria	*Roseiarcus*
	Planctomycetes	*SM1A02*
Plant_growth_promoter	Actinobacteria	*Amycolatopsis*
	Proteobacteria	*Brevundimonas*
	Proteobacteria	*Caulobacter*
	Proteobacteria	*Halomonas*
	Bacteroidetes	*Mucilaginibacter*
	Actinobacteria	*Nocardia* *
	Firmicutes	*Paenibacillus*
	Proteobacteria	*Sphingomonas*
Plant_Pathogenic	Firmicutes	*Candidatus Phytoplasma*
	Firmicutes	*Clostridium sensu stricto 1*
	Proteobacteria	*Dyella*
	Firmicutes	*Mycoplasma*
	Proteobacteria	*Rhizobacter*
Polysaccharide_degrader	Proteobacteria	*Sphingobium*
Root_knot_nematode_associated	Proteobacteria	*Pseudomonas*
	Proteobacteria	*Rhodoplanes*
Soybean_cyst_associated	Proteobacteria	*Polaromonas*
Suppressive_soils	Actinobacteria	*Actinophytocola*
	Actinobacteria	*Actinospica*
	Actinobacteria	*Kribbella*
	Proteobacteria	*Massilia*
	Bacteroidetes	*Niastella*
	Bacteroidetes	*Ohtaekwangia*
	Proteobacteria	*Steroidobacter* **
	Proteobacteria	*Variovorax*

All of the untagged genera were found in both the field and greenhouse populations. * Bacterial genera in field but not in greenhouse populations. ** Bacterial genera in greenhouse but not in field populations.

## Data Availability

The original contributions presented in this study are included in the article/supplementary material. Further inquiries can be directed to the corresponding author.

## Supplementary Materials

The following supporting information can be downloaded at https://www.mdpi.com/article/10.3390/microorganisms13030487/s1; Figure S1: The location of 15 sampled agricultural fields showing *M. hapla* occurrence (present [Mh+] and absence [Mh-]), soil group (mineral [circle] and muck [diamond]) and soil food web conditions (SFW: degraded [red], disturbed [yellow], stable [green]). Figure S2: Stacked bar plots showing the relative abundance of bacteria genera associated with field (A) and greenhouse (B) *Meloidogyne hapla* populations originating from different soil groups (SG: mineral [white] and muck [brown]) and soil food web conditions (SFW: Deg—degraded [red], Dist—disturbed [yellow]). Colors of the bacterial phyla correspond with colors in the stacked bar plots and each bar represents a population in either the field or greenhouse. The relative abundance of phyla was variable across the field and greenhouse populations. Sequences were assigned to taxonomic groups using the ACT (alignment, classification, tree service; version 1.2.12; https://www.arb-silva.de/aligner/ accessed on 20 January 2022) tool of the SILVA online database. Table S1: Worm lysis buffer (WLB) mix used to extract bacterial DNA associated with *Meloidogyne hapla* populations isolated directly from field soils and greenhouse cultures. Table S2: Polymerase chain reaction (PCR) mix with volumes and thermocycle settings (temperature, time, and cycles) used to amplify the 16S region of the bacterial ribosome. Table S3: Taxonomy (phylum, class, order, family and fenera) and functions of the core bacteria associated with the presence or absence of *M. hapla* in the fields from where the nematode populations were collected [1] and their presence or absence in the greenhouse and field populations. Table S4: Taxonomy (phylum, class, order, family and genera) and function of the 25 bacterial indicators of the *M. hapla* absence (OTUs 1–16) or presence (OTUs 17–25) in soils from where the *M. hapla* populations were isolated [1] and their presence or absence in nematodes from the greenhouse and/or field populations.
