# Supplementary material for: The Composition and Function of Bacterial Communities Associated with the Northern Root-Knot Nematode (Meloidogyne hapla) Populations Showing Parasitic Variability
Source: Microorganisms. 2025 Feb 22;13(3):487. doi: 10.3390/microorganisms13030487 (PMC11946340; doi:10.3390/microorganisms13030487)
Supplement: Supplementary file 1 [file microorganisms-13-00487-s001.zip › microorganisms-3412354-supplementary.pdf]

Supplementary Materials

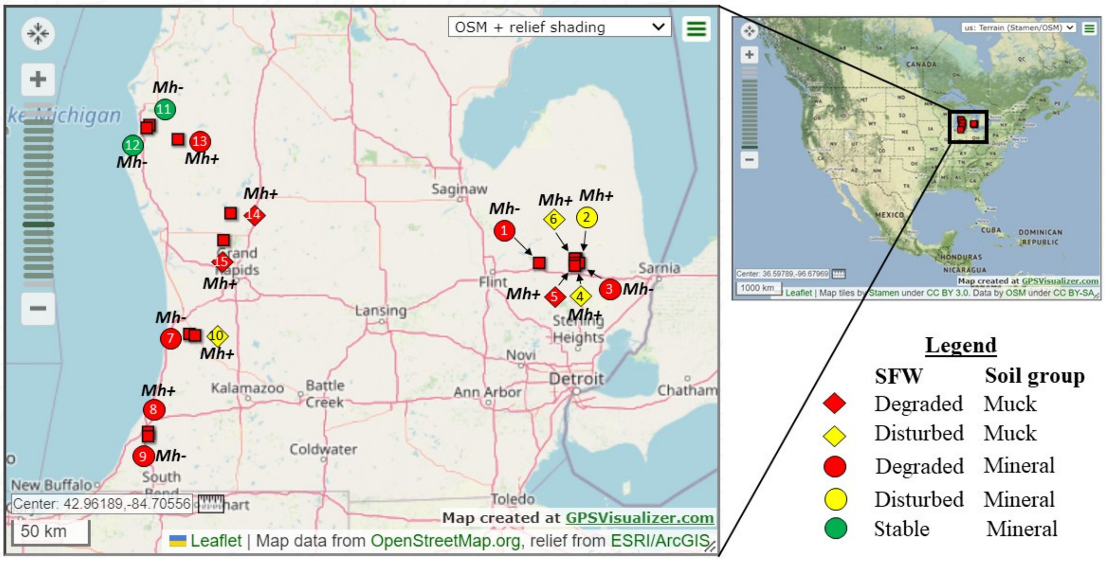

Figure S1: Location of 15 sampled agricultural fields showing *M. hapla* occurrence (Present [Mh+] and Absence [Mh-]), soil group (Mineral [circle] and Muck [diamond]) and soil food web conditions (SFW: Degraded [red], Disturbed [yellow], Stable [green])

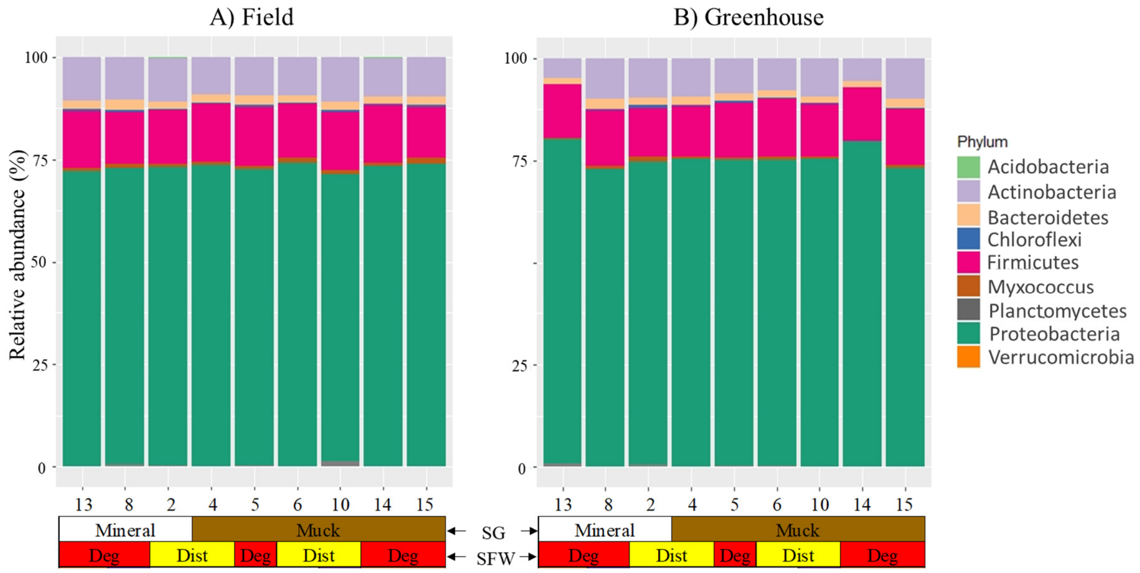

Figure S2 | Stacked bar plots showing the relative abundance of bacteria genera associated with field (A) and greenhouse (B) *Meloidogyne hapla* populations originating from different soil groups (SG: Mineral [white] and Muck [brown]) and soil food web conditions (SFW: Deg-Degraded [red], Dist-Disturbed [yellow]). Colors of bacterial phyla correspond with colors in the stacked bar plots and each bar represents a population in either field or greenhouse. Relative abundance of phyla was variable across the field and greenhouse populations. Sequences were assigned to taxonomic groups using the ACT (alignment, classification, tree service; https://www.arb-silva.de/aligner/) tool of SILVA online database.

Table S1: Worm lysis buffer (WLB) mix used to extract bacterial DNA associated with *Meloidogyne hapla* populations isolated directly from field soils and greenhouse cultures.

**WLB Mix**	**µL**
1M KCL	500
1M Tris pH 8.3	100
1M $MgCl_2$	25
NP40	45
Tween 20	45
2% Gelatin	50
dd H_20	9,235
**Total**	**10,000**

Table S2: Polymerase chain reaction (PCR) mix with volumes and thermocycle settings (temperature, time and cycles) used to amplify the 16S region of the bacterial ribosome.

PCR MIX

Step 1

**PCR Mix**	**μL**	**Total (μL)**
Dream Taq	6.25	937.5
ITS1F	0.375	60
ITS2R	0.375	60
H_20	2	320
BSA	2	320
DNA	1	

Step 2

**PCR Mix**	**μL**	**Total (μL)**
Dream Taq	6.25	1000
ITS1F (with frameshift)	0.375	60
ITS2R (with frameshift)	0.375	60
H_20	1	160
BSA	2	320
DNA	2 from step1	

THERMOCYCLE

Step 1

**Temp (°C)**	**Time(min)**	**Cycles**
95	5:00	
95	0:30	10x
50	0:30	
72	1:00	
72	7:00	
12	inf	

Step 2

**Temp (°C)**	**Time(min)**	**Cycles**
95	5:00	
95	0:30	10x
50	0:35	
72	1:05	
72	7:00	
12	inf	

Table S2 (cont'd)

Step 3		
**PCR Mix**	**μL**	**Total (μL)**
Dream Taq	8	1280
PCR F	0.5	80
H_20	0.5	80
Barcode	1	
DNA	4 from step2	

Step 3		
**Temp (°C)**	**Time(min)**	**Cycles**
95	5:00	
95	0:40	
63	0:50	10x
72	7:00	
72	7:00	
12	inf	

Table S3: Taxonomy (Phylum, Class, Order, Family and Genera) and functions of core-bacteria associated with presence or absence of *M. hapla* in fields from where the nematode populations were collected [1] and their presence or absence in the greenhouse and field populations.

Phyla	Class	Order	Family	Genera	Functional group
Actinobacteria	Actinobacteria	Micrococcales	Micrococcaceae	*Arthrobacter*	Nematicidal
	Actinobacteria	Micromonosporales	Micromonosporaceae	*Dactylosporangium*	Nematicidal
Bacteroidetes	Cytophagia	Cytophagales	Hymenobacteraceae	*Adhaeribacter*	Other
Firmicutes	Bacilli	Paenibacillales	Paenibacillaceae	*Paenibacillus*[1]	Plant_growth_promoter
	Erysipelotrichia	Erysipelotrichales	Turicibacteraceae	*Turicibacter*	Other
Proteobacteria	Alphaproteobacteria	Hyphomicrobiales	Methylobacteriaceae	*Balneimonas*	Other
	Alphaproteobacteria	Rhizobiales	Devosiaceae	*Devosia*[2]	Nematicidal
	Alphaproteobacteria	Sphingomonadales	Sphingomonadaceae	*Kaistobacter*	Enhanced nematode parasitism
	Alphaproteobacteria	Rhodospirillales	Reyranellaceae	*Reyranella*	Soybean cyst associated
	Alphaproteobacteria	Rhizobiales	Xanthobacteraceae	Rhodoplanes[3]	Root_knot_nematode_associated
	Alphaproteobacteria	Sphingomonadales	Sphingomonadaceae	Sphingobium[4]	Polysaccharide-degrader

[1] Bacteria present in all field and greenhouse *M. hapla* Populations

[2] Bacteria present in field *M. hapla* Populations 10 and 13, and all greenhouse *M. hapla* Populations except in Population 13

[3] Bacteria present in all field *M. hapla* Populations, and greenhouse *M. hapla* Populations 2, 5, 10, 4 and 8

[4] Bacteria present in field *M. hapla* Population 2

List of indicators adopted from a previous study by Lartey et al.[1].

Table S4: Taxonomy (Phylum, Class, Order, Family and Genera) and function of 25 bacterial indicators of *M. hapla* absence (OTUs 1 – 16) or presence (OTUs 17 – 25) in soils from where *M. hapla* populations were isolated [1] and their presence or absence in nematodes from the greenhouse and/or field populations.

**Phylum**	**Class**	**Order**	**Family**	**Genera**	**Functional group**
Acidobacteria	Acidobacteria	Acidobacteriales	Acidobacteriaceae	*Chloracidobacteria sp. (OTU2)*	Nematicidal
	Solibacteres	Solibacterales		*Solibacterales sp. (OTU10)*	Plant growth promoter
Actinobacteria				*Actinobacteria sp. (OTU14)*	Nematicidal
				Actinobacteria sp. (OTU18)	Nematicidal
	Actinobacteria	Micrococcales	Intrasporangiaceae	*Phycicoccus sp. (OTU19)*	Other
	Actinobacteria	Micromonosporales	Micromonosporaceae	*Actinoplanes sp. (OTU20)*	Suppressive soils
	Thermoleophilia	Solirubrobacterales		*Solirubrobacterales sp. (OTU21)*	Nematicidal
	Thermoleophilia	Gaiellales	Gaiellaceae	*Gaiellaceae sp. (OTU23)*	Other
Bacteroidetes	Chitinophagia	Chitinophagales	Chitinophagaceae	*Flavitalea populi (OTU5)*	Plant pathogenic
	Sphingobacteriia	Sphingobacteriales		*Sphingobacteriales sp. (OTU12)*	Root knot nematode associated
Chloroflexi				*Chloroflexi sp. (OTU17)*	Nematicidal
				Chloroflexi sp. (OTU24)	Nematicidal

Gemmatimonadetes	Gemmatimonadetes	Gemmatimonales	Gemmatimonaceae	*Gemmatimonas sp. (OTU4)*	Suppressive soils
Planctomycetes	Planctomycetia	Pirellulales	Pirellulaceae	*Pirellulaceae sp. (OTU22)*	Other

Table S4 (cont'd)

**Phylum**	**Class**	**Order**	**Family**	**Genera**	**Functional group**
Proteobacteria	Deltaproteobacteria	Myxococcales	Sorangium	*Sorangium sp. (OTU1)*	Polysaccharide degrader
	Alphaproteobacteria	Hyphomicrobiales	Methylobacteriaceae	*Balneimonas sp. (OTU3)*	Suppressive soils
	Alphaproteobacteria	Hyphomicrobiales	Rhizobiaceae	*Rhizobium sp. (OTU6)* [1]	Nitrogen fixer
	Alphaproteobacteria	Sphingomonadales	Sphingomonadaceae	*Kaistobacter sp. (OTU7)*	Enhanced nematode parasitism
	Betaproteobacteria	Burkholderiales	Oxalobacteraceae	*Oxalicibacterium sp. (OTU8)*	Other
	Alphaproteobacteria	Caulobacterales	Caulobacteraceae	*Brevundimonas sp. (OTU9)*[2]	Plant_growth_promoter
	Alphaproteobacteria	Rhodospirillales	Azospirillaceae	*Skermanella sp. (OTU11)*	Other
	Betaproteobacteria	Rhodocyclales	Zoogloeaceae	*Thauera sp. (OTU13)*	Other
	Deltaproteobacteria	Syntrophobacterales	Syntrophobacteraceae	*Syntrophobacteraceae sp. (OTU15)*	Other
	Alphaproteobacteria	Hyphomicrobiales	Afifellaceae	*Afifella sp. (OTU25)*	Root knot nematode associated

Verrucomicrobiota	Verrucomicrobiae	Pedosphaerales	*Pedosphaerales sp. (OTU16)*	Other

[1]Bacteria present in all field *M. hapla* populations, and all greenhouse *M. hapla* populations with the exception of greenhouse Population 14.

[2]Bacteria present in field *M. hapla* Populations 8, and greenhouse *M. hapla* Population 8 and 2.

List of indicators adopted from a previous study by Lartey et al.[1]
